# DAS-28-based EULAR response and HAQ improvement in rheumatoid arthritis patients switching between TNF antagonists

**DOI:** 10.1186/1471-2474-10-91

**Published:** 2009-07-23

**Authors:** Federico Navarro-Sarabia, Dolores Ruiz-Montesinos, Blanca Hernandez, Victoria Navarro-Compán, Sara Marsal, Mireia Barcelo, Eva Perez-Pampín, Juan J Gómez-Reino

**Affiliations:** 1 Rheumatology Service, Hospital Universitario Virgen Macarena, Sevilla, Spain; 2 Rheumatology Service, Hospital Universitario Vall d'Hebron, Barcelona, Spain; 3 Rheumatology Service, Hospital Clínico de Santiago and Department of Medicine, USC, Santiago, Spain

## Abstract

**Introduction:**

No definitive data are available regarding the value of switching to an alternative TNF antagonist in rheumatoid arthritis patients who fail to respond to the first one. The aim of this study was to evaluate treatment response in a clinical setting based on HAQ improvement and EULAR response criteria in RA patients who were switched to a second or a third TNF antagonist due to failure with the first one.

**Methods:**

This was an observational, prospective study of a cohort of 417 RA patients treated with TNF antagonists in three university hospitals in Spain between January 1999 and December 2005. A database was created at the participating centres, with well-defined operational instructions. The main outcome variables were analyzed using parametric or non-parametric tests depending on the level of measurement and distribution of each variable.

**Results:**

Mean (± SD) DAS-28 on starting the first, second and third TNF antagonist was 5.9 (± 2.0), 5.1 (± 1.5) and 6.1 (± 1.1). At the end of follow-up, it decreased to 3.3 (± 1.6; Δ = -2.6; p > 0.0001), 4.2 (± 1.5; Δ = -1.1; p = 0.0001) and 5.4 (± 1.7; Δ = -0.7; p = 0.06). For the first TNF antagonist, DAS-28-based EULAR response level was good in 42% and moderate in 33% of patients. The second TNF antagonist yielded a good response in 20% and no response in 53% of patients, while the third one yielded a good response in 28% and no response in 72%. Mean baseline HAQ on starting the first, second and third TNF antagonist was 1.61, 1.52 and 1.87, respectively. At the end of follow-up, it decreased to 1.12 (Δ = -0.49; p < 0.0001), 1.31 (Δ = -0.21, p = 0.004) and 1.75 (Δ = -0.12; p = 0.1), respectively. Sixty four percent of patients had a clinically important improvement in HAQ (defined as ≥ -0.22) with the first TNF antagonist and 46% with the second.

**Conclusion:**

A clinically significant effect size was seen in less than half of RA patients cycling to a second TNF antagonist.

## Background

Treatment with TNF antagonists has improved the outcome of rheumatoid arthritis (RA) patients [1]. In both early and established RA, two-thirds of patients achieve meaningful clinical responses, yet one-third do not respond. Additionally, a number of patients initially responding develop acquired drug resistance or gradual drug failure, and some have to discontinue the biologic treatment due to adverse events. Overall, the 3-year retention rate of TNF antagonists in RA is around 65% [2].

TNF antagonists as a group have similar efficacy in RA, although their effectiveness differs in other rheumatic diseases. Moreover, case series and nonrandomized, open-label observational studies in RA indicate that some patients may fail to respond to one TNF inhibitor but will respond to another. This is partially supported by data showing that TNF antagonists differ in their pharmacokinetics and mechanisms of action [3]. Nevertheless, there are no definitive data regarding the value of switching between TNF antagonists. Another therapeutic option is to switch to a different class of biologic agent such as rituximab, tocilizumab or abatacept [4-6].

The aim of this study was to evaluate in a clinical setting the clinical response based on evaluation of HAQ and EULAR response criteria in RA patients with an insufficient response or loss of efficacy to the first TNF antagonist who were switched to a second or third one.

## Methods

This was an observational, prospective study of a cohort of 417 RA patients treated with TNF antagonists in three university hospitals in Spain between January 1999 and December 2005. A database was created at the participating centres, with well-defined operational instructions. Patients who had participated in clinical trials were excluded.

Patients had been systematically evaluated at the initiation of therapy and every three months thereafter. Patients switching between TNF antagonists or switching to rituximab were evaluated on starting therapy and every 3 months thereafter. Evaluations included painful and swollen joint counts, visual analogue scales of pain, global health assessment by the patient and the physician, ESR, C-reactive protein (CRP), Health Assessment Questionnaire (HAQ) and DAS-28 score. DAS-28-based EULAR response was estimated. Data on the reason for switching to a second TNF antagonist were recorded.

Descriptive statistics with central tendency and dispersion measures were calculated. The main outcome variables were analyzed using parametric or non-parametric tests depending on the level of measurement and distribution of each variable. A p-value < 0.05 (two tailed) was considered significant. Survival analysis was performed using Kaplan-Meyer curves.

The study was conducted according to good clinical practice as applicable to epidemiological studies, which ensures that the design, implementation and communication of data are reliable, and that patients' rights, integrity and data confidentiality are protected. The study protocol was approved by the Ethics Committee of the Hospital Universitario Virgen Macarena which considered that informed consent was not required due to the retrospective nature of the analysis of anonymous data.

## Results

The initial TNF antagonist was infliximab (INF) in 238 cases (57%), etanercept (ETA) in 141 (34%), and adalimumab (ADA) in 38 (9%). Eighty-three patients had switched to a second TNF antagonist and 18 to a third TNF antagonist. Mean patient follow-up was 21.4 + 15.6 months, and TNF exposure was 443 patient-years for INF, 200.2 patient-years for ETA and 31.7 patient-years for ADA. Switching in 48 cases (58%) was due to inefficacy, in 24 cases (29%) to adverse events, and in 11 cases (13%) to other reasons, primarily the doctor's or patient's decision.

Relevant clinical data on the 417 patients (82% women) are presented in table 1. The mean age of patients starting their first TNF antagonist was 53 ± 13 years, and mean disease duration was 10.4 ± 8.2 years. Sixty-eight percent were RF positive and 74% presented erosions. Three hundred ninety-six patients (94%) received concomitant DMARD; 324 methotrexate (MTX), 33 leflunomide (LF) and 21 antimalarials. During follow up, 263 patients (63%) continued the first TNF antagonist, 83 (20%) switched to a second, and 18 (4%) to a third. Seventeen percent of patients received no other biologic after discontinuation. Forty-six patients treated with a second TNF antagonist had switched from INF or ADA to ETA, 25 from INF to ADA and 12 from ETA to ADA. Among patients treated with a third TNF antagonist, 12 had changed from ETA to ADA, 5 from ADA to ETA, and 1 from ETA to INF.

**Table 1:** Characteristics of patients switching between TNF antagonists

	1st TNF antagonist (417)	2nd TNF antagonist (83)	3rd TNF antagonist (18)
Age, years (SD)	53 ± 13	52 ± 12	44 ± 11
Gender (F)	342 (82%)	68(82%)	17(94%)
Disease duration years (SD)	10.4 (± 8,2)	10 (± 8)	11(± 8)
RF positive, %	68%	69%	61%
Radiographic erosions, %	74%	79%	94%
Concomitant DMARD, %	94%	94%	100%
Methotrexate	78%	71%	50%
Leflunomide	8%	2%	17%
Antimalarial	5%	4%	5%
Oral glucocorticoids, %	83%	83%	51%
Adalimumab, n	38	36	12
Etanercept, n	141	46	5
Infliximab, n	238	1	1

Mean DAS-28 on starting the first TNF antagonist was 5.9 ± 2.0 and decreased to 3.3 ± 1.6, at the end of follow-up. The improvement was statistically significant (Δ = -2.6; p > 0.0001) for the whole group as well as for the three TNF antagonists considered independently. DAS-28 on starting the second TNF antagonist was 5.1 ± 1.5 and decreased to 4.2 ± 1.5 at the end of follow-up (Δ = -1.1; p = 0.0001). DAS-28 on starting the third TNF antagonist was 6.1 ± 1.1 and decreased to 5.4 ± 1.7 at the end of follow-up (Δ = -0.7; p = 0.06). The results are shown in table 2.

**Table 2:** Improvement in DAS 28 and HAQ, and DAS-28-based EULAR response in patients switching between TNF antagonists

	1st TNF antagonist				2nd TNF antagonist				3rd TNF antagonist			
	Initial	Final	Δ	p	Initial	Final	Δ	p	Initial	Final	Δ	P
DAS-28	5.9	3.3	-2.6	<0.0001	5.1	4.2	-1.1	0.0001	6.1	5.4	-0.7	0.06
HAQ	1.61	1.12	-0.49	<0.0001	1.52	1.31	-0.21	<0.004	1.87	1.75	-0.12	0.1
Good (%)*		174(42)				17(20)				5(28)		
Moderate (%)*		138(33)				20(27)						
No response (%)*		105(25)				44(53)				13(72)		

* DAS-28-based EULAR response

The DAS-28-based EULAR response level for the first TNF antagonist was good in 42% of patients and moderate in 33%. For the second anti-TNF, good response was achieved in 20% of cases and 53% failed to respond. For the third TNF antagonist, 26% of cases had a good response and 72% failed to respond (Table 2). Response to the second TNF antagonist did not differ significantly (p = 0.5) by the reason for switching (Table 3).

**Table 3:** DAS-28-based EULAR response in patients switchingto a second TNF antagonist, by reason for switching

	Inefficacy N (%)	Adverse events N (%)	Other reasons N (%)
Good	10 (20)	6 (25)	1 (10)
Moderate	12 (25)	8 (30)	2 (18)
No response	26 (55)	10 (45)	8 (72)

**Figure 1:**
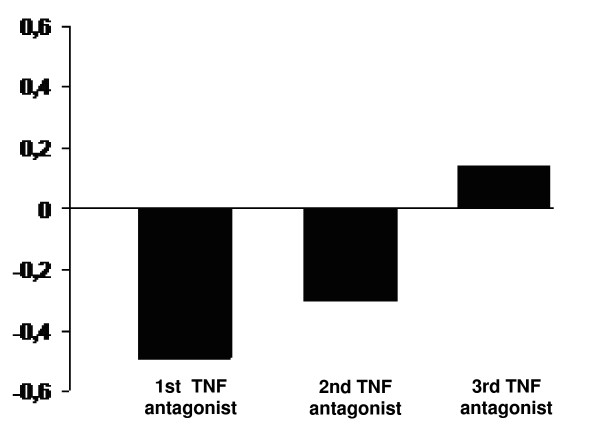
Mean cumulative change in HAQ from starting therapy in patients with rheumatoid arthritis following cycling between TNF antagonists.

HAQ improved significantly (Table 2) with use of the first TNF antagonist, with a mean baseline score of 1.61 and final score of 1.12 (Δ = -0.49; p < 0.0001), with 64% of patients showing an improvement ≥ -0.22. Improvement was not significantly different with the three biologics. For the second TNF antagonist, mean initial and final HAQ were 1.52 and 1.31, respectively (Δ = -0.21, p < 0.004), with 46% of patients showing an improvement ≥ -0.22. For the third TNF antagonist, HAQ scores were 1.87 and 1.75 at the initial and final evaluation, respectively (Δ = -0.12; p = 0.1). The mean cumulative change in HAQ from pre-treatment with the first TNF antagonist is depicted in figure 1.

Retention rates with the first TNF antagonist were 80%, 62%, 53% and 34% at 12, 24, 36 and 60 months, respectively. No significant differences were found among the three drugs (p = 0.1). The reasons for discontinuation were inefficacy (40%), adverse events (40%) and other (20%). Retention rates with the second TNF antagonist were 70%, 60% and 47% at 12, 24 and 36 months, respectively. Twenty-five patients discontinued the biologic, most commonly due to inefficacy (77%). Only 9 of the 18 patients switching to a third TNF antagonist retained the biologic at 6 months. Six stopped the biologic due to inefficacy.

Fifty-four patients had a severe adverse event during treatment with the first TNF antagonist. Infusion reaction was the most frequent adverse event, occurring in 16 patients treated with INF, followed by urticaria or severe skin rash in 9 patients, and upper respiratory tract infection in 8. Other less frequent adverse events were congestive heart failure, tuberculosis, herpes zoster infection, acute pancreatitis, cutaneous vasculitis and lupus-like syndrome. Seven patients had severe adverse events while treated with the second TNF antagonist (Table 4).

**Table 4:** Serious adverse events occurring in patients switching between TNF antagonists

	1st TNF antagonist (n = 417)	2nd TNF antagonist (n = 83)	3rd TNF antagonist (n = 18)
Upper respiratory infections	8	1	
Urticaria	3		
Systemic lupus erythematosus	2		
Herpes zoster	2		
Rash	3	1	1
Tuberculosis	2	1	
Infusion reactions	16	1	
Cardiac insufficiency	3		
Acute pancreatitis	2		
Cutaneous vasculitis	2		
Erythema	3		
Infectious arthritis		2	
Leukopenia		1	

## Discussion

In the present study we describe relevant outcomes in clinical practice in RA patients failing to respond to one TNF antagonist and switching to another, in comparison with RA patients who retain the first antagonist. Our data show that a number of patients switching between TNF antagonists may attain a significant response, yet a large proportion of patients have marginal or no clinical improvement. Improvement is generally negligible in patients switching to a third biologic. Interestingly, patients switching to a third TNF antagonist were younger, more frequently female, and had a higher prevalence of erosions despite the fact that disease duration was similar to the other patients. This group probably represents a subset of patients with more severe disease which is resistant to these biologics.

Up to September 2008, there were 35 reports in the literature [7-42] on switching between TNF antagonists in RA patients who experience treatment failure. Twenty reports were based on observational studies, 10 on case series and 6 on prospective cohorts from biologic registries. Additionally there were 2 randomized clinical trials [8,11]: 1 double-blind and 1 open-label study. The two clinical trials demonstrate the efficacy of switching between TNF antagonists. On the whole, the numerous design limitations of the other studies limit their value. Nevertheless, the vast majority of them report on the favourable efficacy of switching, yet information on the effect size is largely unreported.

Most patients included in previous publications were women (83%, range: 60–100), with an average age of 52 years (range: 32–68), and a disease duration of 12 years (range: 3–27). When reported at baseline, mean DAS was 5.6 (range: 2.4–6.8), and mean HAQ 1.7 (range: 1.5–1.9). This baseline information is no different from what was found in our study. Of note, few publications report on DAS-28 effect size, DAS-based EULAR response and HAQ improvement in patients switching between TNF antagonists in comparison with patients who retain the first antagonist. A value of -0.22 in HAQ is considered the minimum clinically important difference (MCID) in studies of responsiveness [43]. The size of improvement with the first TNF antagonist (-0.40) is within the range of what has been reported in clinical trials [44-46]. Two-thirds of patients treated with the first and less than half of those treated with the second TNF antagonist had a MCID in HAQ. Despite long-standing disease, HAQ improvement was parallel to DAS-28 improvement, a result that was unanticipated based on previous data [47].

Three studies [14,15,20] have described retention rates of a second TNF antagonist as a surrogate for effectiveness. Overall, the probability of retaining a second TNF antagonist was lower than retaining the first one. The probability was influenced by the reason for drug replacement, i.e. drug failure or adverse event. Interestingly, the reasons for stopping a second drug were related to the reasons for stopping the first drug [15]. Although the retention rate of a drug can be taken as a reasonable indicator of its effectiveness, parameters other than efficacy and safety, such as co-morbidity, co-medications, costs, availability of other therapies, patients' and physicians' expectations, and adherence to treatment could influence drug survival. In our study, retention rates of the second and third TNF antagonists were within the boundaries of what other authors have reported, suggesting that these rates are consistently found in clinical practice. Herein, we show that lack of response to a first TNF antagonist does not predict the response to a second one, yet the efficacy of a second TNF antagonist is inferior to that of the first.

## Conclusion

A significant treatment effect size with a second TNF antagonist in RA patients failing to respond to the first one is limited to less than half of those treated. Furthermore, RA patients who are refractory to TNF-antagonist treatment have a poorer response to rituximab, abatacept and tocilizumab than patients who are naive to biological drugs. Evidence that the efficacy of cycling between TNF antagonists is similar or superior to switching to the new biologics is largely missing. The results reported by other authors [48] as well as those of our own study suggest that this may not be the case.

## Abbreviations

ADA: Adalimumab; DAS: Disease Activity Score; ETA: Etanercept; EULAR: European League Against Rheumatism; HAQ: Health Assessment Questionnaire; INF: Infliximab; LF: Leflunomide; MCID: Minimum Clinically Important Difference; MTX: Methotrexate; RA: Rheumatoid Arthritis; TNF: Tumor Necrosis Factor.

## Competing interests

DRM, BH, VNC, SM and MB declare that they have no competing interests. FNS and JJGR have participated in Advisory Boards and received lecture fees from Abbott, Bristol-Mayer-Squib, Roche, Schering-Plough and Wyeth.

## Authors' contributions

FNS and JJGR conceived, designed and coordinated the study, and prepared the draft of the manuscript. DRM, VNC, SM and MB collected the data and reviewed the data analyses. BH performed the statistical analysis and contributed to the design of the study. All authors read and approved the final manuscript.

## Pre-publication history

The pre-publication history for this paper can be accessed here:



## References

[B1] Keystone EC (2006). Strategies to control disease in rheumatoid arthritis with tumor necrosis factor antagonists–an opportunity to improve outcomes. Nature Clin Pract Rheumatol.

[B2] Carmona L, Gómez-Reino JJ, BIOBADASER Group (2006). Survival of TNF antagonists in spondylarthritis is better than in rheumatoid arthritis. Data from the Spanish registry BIOBADASER. Arthritis Res Ther.

[B3] Tracey D, Klareskog L, Sasso EH, Salfeld JG, Tak PP (2008). Tumor necrosis factor antagonist mechanisms of action: a comprehensive review. Pharmacol Ther.

[B4] Cohen SB, Emery P, Greenwald MW, Dougados M, Furie RA, Genovese MC, Keystone EC, Loveless JE, Burmester GR, Cravets MW, Hessey EW, Shaw T, Totoritis MC (2006). Rituximab for rheumatoid arthritis refractory to anti-tumor necrosis factor therapy: Results of a multicenter, randomized, double-blind, placebo-controlled, Phase III trial evaluating primary efficacy and safety at twenty-four weeks. Arthritis Rheum.

[B5] Emery P, Keystone E, Tony HP, Cantagrel A, van Vollenhoven R, Sanchez A, Alecock E, Lee J, Kremer J (2008). IL-6 receptor inhibition with tocilizumab improves treatment outcomes in patients with rheumatoid arthritis refractory to anti-tumour necrosis factor biologicals: results from a 24-week multicentre randomised placebo-controlled trial. Ann Rheum Dis.

[B6] Genovese MC, Schiff M, Luggen M, Becker JC, Aranda R, Teng J, Li T, Schmidely N, Le Bars M, Dougados M (2008). Efficacy and safety of the selective co-stimulation modulator abatacept following 2 years of treatment in patients with rheumatoid arthritis and an inadequate response to anti-tumour necrosis factor therapy. Ann Rheum Dis.

[B7] Hyrich KL, Lunt M, Dixon WG, Watson KD, Symmons DP, BSR Biologics Register (2008). Effects of switching between anti-TNF therapies on HAQ response in patients who do not respond to their first anti-TNF drug. Rheumatology (Oxford).

[B8] Smolen J, Kay J, Doyle MK, Landewe R, Matteson EL, Wollenhaupt J, Gaylis N, Murphy F, Neal J, Zamani O, Zhou Y, Visvanathan S, Hsia EC, Rahman MU (2008). Golimumab, a new human anti-TNF-alpha monoclonal antibody, subcutaneously administered every 4 weeks in patients with active rheumatoid arthritis who were previously treated with anti-TNF-alpha agent(s): Results of the randomized double-blind, placebo-c (sic) [abstract]. Ann Rheum Dis.

[B9] Karlsson JA, Kristensen LE, Kapetanovic MC, Gulfe A, Saxne T, Geborek P (2008). Treatment response to a second or third TNF-inhibitor in RA: results from the South Swedish Arthritis Treatment Group Register. Rheumatology (Oxford).

[B10] Blom M, Kievit W, Fransen J, Kuper IH, Laar M van der, de Rooj D, de Gendt C, Jansen TL (2007). Effectiveness of a switch to a second anti-TNF-α in primary nonresponders, secondary nonresponders and failure due to adverse events. [abstract]. Arthritis Rheum.

[B11] Furst DE, Gaylis N, Bray V, Olech E, Yocum D, Ritter J, Weisman M, Wallace DJ, Crues J, Khanna D, Eckel G, Yeilding N, Callegari P, Visvanathan S, Rojas J, Hegedus R, George L, Mamun K, Gilmer K, Troum O (2007). Open-label, pilot protocol of patients with rheumatoid arthritis who switch to infliximab after an incomplete response to etanercept: The OPPOSITE study. Ann Rheum Dis.

[B12] Finckh A, Ciurea A, Brulhart L, Kyburz D, Möller B, Dehler S, Revaz S, Dudler J, Gabay C (2004). Anti-tumor necrosis factor alpha switching in rheumatoid arthritis and juvenile chronic arthritis. Arthritis Rheum.

[B13] Buch MH, Bingham SJ, Bejarano V, Bryer D, White J, Reece R, Quinn M, Emery P (2007). Therapy of patients with rheumatoid arthritis: outcome of infliximab failures switched to etanercept. Arthritis Rheum.

[B14] Conti F, Ceccarelli F, Marocchi E, Magrini L, Romana Spinelli F, Spadaro A, Scrivo R, Valesini G (2007). Switching TNF{alpha} antagonists in patients with ankylosing spondylitis and psoriatic arthritis: an observational study over a five-year period. Ann Rheum Dis.

[B15] Hjardem E, Ostergaard M, Pødenphant J, Tarp U, Andersen LS, Bing J, Peen E, Lindegaard HM, Ringsdal VS, Rødgaard A, Skøt J, Hansen A, Mogensen HH, Unkerskov J, Hetland ML (2007). Do rheumatoid arthritis patients in clinical practice benefit from switching from infliximab to a second tumor necrosis factor alpha inhibitor?. Ann Rheum Dis.

[B16] Hyrich KL, Lunt M, Watson KD, Symmons DP, Silman AJ (2007). Outcomes after switching from one anti-tumor necrosis factor alpha agent to a second anti-tumor necrosis factor alpha agent in patients with rheumatoid arthritis: results from a large UK national cohort study. Arthritis Rheum.

[B17] Iannone F, Trotta F, Montecucco C, Giacomelli R, Galeazzi M, Matucci-Cerinic M, Ferri C, Cutolo M, Maria Bambara L, Triolo G, Ferraccioli G, Valentini G, Lapadula G, GISEA (Gruppo Italiano per lo Studio delle Early Arthritis) (2007). Etanercept maintains the clinical benefit achieved by infliximab in patients with rheumatoid arthritis who discontinued infliximab because of side effects. Ann Rheum Dis.

[B18] Atzeni F, Sarzi-Puttini P, Antivalle M, Turiel M, Carrabba M (2006). Adalimumab in severe rheumatoid arthritis after failure of two anti-TNF agents: a prospective 1-year follow-up study of 15 patients. Ann Rheum Dis.

[B19] Bombardieri S, McKenna F, Drosos AA, Michel BA, Hartz D, Oezer U (2006). Efficacy and safety of adalimumab (HUMIRA^®^) in 899 patients with rheumatoid arthritis who previously failed etanercerpt and/or infliximab in clinical practice [abstract]. Ann Rheum Dis.

[B20] Cantini F, Niccoli L, Benucci M, Chindamo D, Nannini C, Olivieri I, Padula A, Salvarini C (2006). Switching from infliximab to once-weekly administration of 50 mg etanercept in resistant or intolerant patients with ankylosing spondylitis: results of a fifty-four-week study. Arthritis Rheum.

[B21] Gomez-Reino JJ, Carmona L (2006). Switching TNF antagonists in patients with chronic arthritis: an observational study of 488 patients over a four-year period. Arthritis Res Ther.

[B22] Nikas SN, Voulgari PV, Alamanos Y, Papadopoulos CG, Venetsanopoulou AI, Georgiadis AN, Drosos AA (2006). Efficacy and safety of switching from infliximab to adalimumab: a comparative controlled study. Ann Rheum Dis.

[B23] Solau-Gervais E, Laxenaire N, Cortet B, Dubucquoi S, Duquesnoy B, Flipo RM (2006). Lack of efficacy of a third tumour necrosis factor alpha antagonist after failure of a soluble receptor and a monoclonal antibody. Rheumatology (Oxford).

[B24] Bingham CO, Haraoui PB, Rigby WFC, Montalvo-Lugo V, Chon T (2005). Disease characteristics of patients with rheumatoid arthritis that failed to respond to infliximab and the response to etanercept therapy: preliminary data from EMBARK study. Ann Rheum Dis.

[B25] Bombardieri S, Ruiz AA, Fardellone P, Geusens P, McKenna F, Unnebrink K, Oezer U, Kary S, Kupper H, Burmester GR (2007). Research in Active Rheumatoid Arthritis (ReAct) Study Group Effectiveness of adalimumab for rheumatoid arthritis in patients with a history of TNF-antagonist therapy in clinical practice. Rheumatology (Oxford).

[B26] Burmester GR, Mariette X, Montecucco C, Monteagudo-Sáez I, Malaise M, Tzioufas AG, Bijlsma JW, Unnebrink K, Kary S, Kupper H (2007). Adalimumab alone and in combination with disease-modifying antirheumatic drugs for the treatment of rheumatoid arthritis in clinical practice: the Research in Active Rheumatoid Arthritis (ReAct) trial. Ann Rheum Dis.

[B27] Cantini F, Niccoli L, Porciello G (2005). Switching from infliximab or adalimumab to etanercept 50 Mg/once weekly in resistant or intolerant patients with rheumatoid arthritis: 24-week results. Arthritis Rheum.

[B28] Cohen G, Courvoisier N, Cohen JD, Zaltni S, Sany J, Combe B (2005). The efficiency of switching from infliximab to etanercept and vice-versa in patients with rheumatoid arthritis. Clin Exp Rheumatol.

[B29] Delaunay C, Farrenq V, Marini-Portugal A, Cohen JD, Chevalier X, Claudepierre P (2005). Infliximab to etanercept switch in patients with spondyloarthropathies and psoriatic arthritis: preliminary data. J Rheumatol.

[B30] Bijl AE Van der, Breedveld FC, Antoni C, Kalden JR, Kary S, Burmester GR, Unnebrink K, Kupper H (2005). Adalimumab (HUMIRA^®^) is effective in treating patients with rheumatoid arthritis who previously failed infliximab treatment. Ann Rheum Dis.

[B31] Wick MC, Ernestam S, Lindblad S, Bratt J, Klareskog L, van Vollenhoven RF (2005). Adalimumab (Humira) restores clinical response in patients with secondary loss of efficacy from infliximab (Remicade) or etanercept (Enbrel): results from the STURE registry at Karolinska University Hospital. Scand J Rheumatol.

[B32] Katsicas MM, Russo RA (2005). Use of infliximab in patients with systemic juvenile idiopathic arthritis refractory to etanercept. Clin Exp Rheumatol.

[B33] Keystone EC, Perruquet JL, Lidman RW (2004). Switching anti-TNF therapy: real-world outcome of patients with rheumatoid arthritis who failed either infliximab or etanercept treatment and switched to another TNF inhibitor [abstract]. Arthritis Rheum.

[B34] Favalli EG, Arreghini M, Arnoldi C, Panni B, Marchesoni A, Tosi S, Pontikaki I (2004). Anti-tumor necrosis factor alpha switching in rheumatoid arthritis and juvenile chronic arthritis. Arthritis Rheum.

[B35] Hansen KE, Hildebrand JP, Genovese MC, Cush JJ, Patel S, Cooley DA, Cohen SB, Gangnon RE, Schiff MH (2004). The efficacy of switching from etanercept to infliximab in patients with rheumatoid arthritis. J Rheumatol.

[B36] Haraoui B, Keystone EC, Thorne JC, Pope JE, Chen I, Asare CG, Leff JA (2004). Clinical outcomes of patients with rheumatoid arthritis after switching from infliximab to etanercept. J Rheumatol.

[B37] Sanmarti R, Gomez-Puerta JA, Rodriguez-Cros JR, Albaladejo C, Munoz-Gomez J, Canete JD (2004). Etanercept in rheumatoid arthritis patients with a poor therapeutic response to infliximab. Med Clin (Barc).

[B38] Yazici Y, Erkan D (2004). Do etanercept-naive patients with rheumatoid arthritis respond better to infliximab than patients for whom etanercept has failed?. Ann Rheum Dis.

[B39] Ang HT, Helfgott S (2003). Do the clinical responses and complications following etanercept or infliximab therapy predict similar outcomes with the other tumor necrosis factor-alpha antagonists in patients with rheumatoid arthritis?. J Rheumatol.

[B40] van Vollenhoven R, Harju A, Brannemark S, Klareskog L (2003). Treatment with infliximab (Remicade) when etanercept (Enbrel) has failed or vice versa: data from the STURE registry showing that switching tumour necrosis factor alpha blockers can make sense. Ann Rheum Dis.

[B41] Brocq O, Plubel Y, Breuil V, Grisot C, Flory P, Mousnier A, Euller-Ziegler L (2002). Etanercept–infliximab switch in rheumatoid arthritis 14 out of 131 patients treated with anti TNFalpha. Presse Med.

[B42] Shergy W, Phillips RM, Hunt RE, Hernandez J (2001). Experience with commercial remicade (infliximab) at a large community-based rheumatology practice [abstract]. Arthritis Rheum.

[B43] Beaton DE, Bombardier C, Katz JN, Wright JG, Wells G, Boers M, Strand V, Shea B (2001). Looking for important change/differences in studies of responsiveness. OMERACT MCID Working Group. J Rheumatol.

[B44] Lipsky PE, Heijde DM van der, St Clair EW, Furst DE, Breedveld FC, Kalden JR, Smolen JS, Weisman M, Emery P, Feldmann M, Harriman GR, Maini RN (2000). Infliximab and methotrexate in the treatment of rheumatoid arthritis. N Engl J Med.

[B45] Weinblatt ME, Keystone EC, Furst DE, Moreland LW, Weisman MH, Birbara CA, Teoh LA, Fischkoff SA, Chartash EK (2003). Adalimumab, a fully human anti-tumor necrosis factor alpha monoclonal antibody, for the treatment of rheumatoid arthritis in patients taking concomitant methotrexate: the ARMADA trial. Arthritis Rheum.

[B46] Weinblatt ME, Kremer JM, Bankhurst AD, Bulpitt KJ, Fleischmann RM, Fox RI, Jackson CG, Lange M, Burge DJ (1999). A trial of etanercept, a recombinant tumor necrosis factor receptor: Fc fusion protein, in patients with rheumatoid arthritis receiving methotrexate. N Engl J Med.

[B47] Aletaha D, Ward MM (2006). Duration of rheumatoid arthritis influences the degree of functional improvement in clinical trials. Ann Rheum Dis.

[B48] Finckh A, Ciurea A, Brulhart L, Kyburz D, Möller B, Dehler S, Revaz S, Dudler J, Gabay C (2007). Physicians of the Swiss Clinical Quality Management Program for Rheumatoid Arthritis: B cell depletion may be more effective than switching to an alternative anti-tumor necrosis factor agent in rheumatoid arthritis patients with inadequate response to anti-tumor necrosis factor agents. Arthritis Rheum.

